# Supplementary material for: Implementing the MATILDA intervention to enhance community participation for older adults with intellectual disability: A feasibility randomised controlled trial and RE-AIM guided process evaluation
Source: PLoS One. 2026 Sep 22;21(9):e0341940. doi: 10.1371/journal.pone.0341940 (PMC13596784; doi:10.1371/journal.pone.0341940)
Supplement: Supplementary file 1 [file pone.0341940.s002.docx]

| **Comment** | **Response** |
| --- | --- |
| **Reviewer 1:** |  |
| 1. The manuscript does not present a clear hierarchy that keeps the primary feasibility aim front and center, because feasibility outcomes, clinical outcomes, process evaluation, and economic elements are all interwoven without a crisp primary feasibility analysis narrative; the authors should tighten the paper around prespecified feasibility endpoints and progression criteria, and keep secondary and exploratory outcomes clearly labelled and clearly subordinated to the feasibility aim. | Thank you for this thoughtful comment. We agree that, in feasibility studies, the primary emphasis should remain on feasibility objectives rather than exploratory clinical outcomes. The primary aim of this study was to assess the feasibility and acceptability of the MATILDA intervention and trial procedures, including recruitment, retention, intervention adherence and outcome data collection. These outcomes remain the primary focus of the manuscript. We have revised the Aim, Results and Discussion sections to more clearly distinguish between feasibility outcomes and exploratory effectiveness outcomes. We have also strengthened the wording throughout the manuscript to emphasise that clinical, process evaluation and economic findings were secondary and exploratory in nature and were included to inform the design of a future definitive trial rather than to evaluate intervention effectiveness. However, given the multifaceted aims of feasibility studies, we believe it is important to retain the reporting of these exploratory outcomes alongside the primary feasibility findings. |
| 2. The blinding description is internally inconsistent, because the trial is described as single blind while the research staff conducting baseline and follow up assessments were not blinded and participant and mentor blinding was not possible; the authors should describe the study as it was conducted, clarify what protections were used to reduce assessment bias, and ensure Methods and Limitations use consistent language. | Thank you for this comment. We agree that the original description of blinding required clarification. Participant and mentor blinding was not possible due to the nature of the intervention, and the research staff conducting baseline and follow-up assessments were also not blinded to treatment allocation. We have therefore revised the Methods section to accurately describe the study procedures and removed any potentially misleading references to the study being single-blind. We have also clarified the steps taken to minimise assessment bias, including the use of standardised outcome measures and study procedures. In addition, we have expanded the Limitations section to acknowledge the potential for assessment bias arising from the lack of blinding. |
| 3. The randomization reporting is incomplete for reproducibility even though stratification by the two sites is stated, because key details such as the allocation concealment mechanism and the permuted block approach with mixed block sizes described in the protocol are not fully carried into the manuscript; the authors should report sequence generation and concealment clearly, including who generated the sequence, how centralized allocation was implemented, and the block structure description at a level that preserves concealment. | Thank you for this comment. We agree that the randomisation procedures should be described in sufficient detail to allow readers to assess the study's methodological rigour. We have therefore expanded the Methods section to provide additional information regarding sequence generation, stratification by study site, allocation concealment, and treatment allocation procedures. Specifically, we now report who generated the allocation sequence, the allocation concealment procedures using opaque sealed envelopes, and the process used to implement treatment assignment. These additions are consistent with the procedures described in the published study protocol and improve the transparency and reproducibility of the trial methods. |
| 4. The two arm design is not used analytically in a way that justifies its inclusion, because the results are primarily descriptive summaries within arms and across time points with limited direct estimation of between arm contrasts; the authors should explain why an active control arm was required for this feasibility question and then report between arm differences for key outcomes at 6 and 12 months, at minimum as estimated effects with 95% confidence intervals. | Thank you for this comment. The primary purpose of this study was to evaluate the feasibility and acceptability of the MATILDA intervention and trial procedures, rather than to assess intervention effectiveness. The inclusion of a control arm was important to determine the feasibility of recruitment, randomisation, retention, outcome measure completion and trial procedures that would be required in a future definitive randomised controlled trial. Consistent with the objectives of a feasibility study, the analyses were primarily descriptive and were not designed or powered to detect between-group differences. To address the reviewer's concern, we have clarified the rationale for including a two-arm randomised design within the Methods and Discussion sections. We have also strengthened the reporting and interpretation of exploratory outcome data by emphasising that these findings should not be interpreted as evidence of effectiveness. While between-group comparisons were not the primary objective of this feasibility study, we have reported exploratory estimates of group differences and associated 95% confidence intervals where appropriate, to inform the design of a future definitive trial. |
| 5. Table 4 and other longitudinal outcome summaries do not provide statistical inference, which makes it difficult for readers to interpret whether observed changes or apparent differences are potentially meaningful versus expected variability in a feasibility sample; the authors should add 95% confidence intervals for within arm change from baseline and for between arm differences at each follow up time point, and they may use a simple baseline adjusted ANCOVA or a repeated measures mixed effect model without overemphasizing p-values. | We thank the reviewer for this suggestion. However, the primary purpose of this study was to assess the feasibility and acceptability of the MATILDA intervention and trial procedures rather than to evaluate intervention effectiveness. Consistent with guidance for pilot and feasibility randomised trials, the study was not powered to detect statistically significant between-group differences in clinical outcomes. We therefore elected to present descriptive summaries of outcome measures over time rather than undertake formal inferential analyses that could be easily overinterpreted. While we agree that confidence intervals can provide information regarding the precision of estimates, we are cautious about presenting effect estimates derived from an underpowered feasibility sample, as these may be unstable and potentially misleading. We have clarified in the manuscript that outcome data are presented descriptively to inform the design and sample size calculations of a future definitive trial. |
| 6. The analysis plan is not presented in a prespecified, verifiable way in the manuscript, because the paper states that no formal statistical tests were performed and does not clearly specify how site stratification is handled in analyses, how missing data are addressed, or how multiple secondary and exploratory outcomes are managed; the authors should either provide a standalone SAP as a supplement or map the protocol statistical methods into the manuscript with enough specificity that the reader can see what was planned versus exploratory. | We thank the reviewer for this comment. The MATILDA study was designed as a feasibility randomised controlled trial, and the statistical analyses were specified within the published study protocol rather than a separate Statistical Analysis Plan. Consistent with feasibility trial methodology, the principal aim was to assess recruitment, retention, intervention adherence, acceptability, and trial procedures rather than effectiveness outcomes. Consequently, analyses were primarily descriptive, and no formal hypothesis testing was planned or undertaken. We agree that the manuscript would benefit from greater clarity regarding the prespecified analytical approach. We have therefore expanded the Methods section to provide additional detail regarding the handling of site stratification, missing data, and secondary and exploratory outcomes. Specifically, we now clarify that outcomes are reported descriptively by study group and assessment time point, that missing data were not imputed, and that secondary outcome measures were included to inform the selection of outcome measures and sample size estimation for a future definitive trial rather than to evaluate treatment effectiveness. We have also included additional information linking the analyses reported in this manuscript to those prespecified in the published protocol. |
| 7. The manuscript describes modifications to at least one instrument during follow-up, which can compromise comparability across time; the authors should specify when changes occurred, how scoring and interpretation remain comparable to baseline, and what sensitivity analyses support longitudinal conclusions under the modified measurement approach. | We thank the reviewer for this comment. The modification referred to was fully documented during the study and is described in the Results section. The purpose of the adaptation was to improve accessibility and comprehension for the mentors rather than to alter the underlying construct being measured. Consistent with the feasibility objectives of the study, outcome data are reported descriptively and interpreted cautiously. We acknowledge that modifications to measurement instruments can potentially affect comparability across time points. However, we do not believe that sensitivity analyses are appropriate in this instance. The study was designed as a feasibility trial and was not powered to undertake inferential analyses of clinical outcomes. Furthermore, the primary aim was not to evaluate intervention effectiveness but to assess the feasibility and acceptability of trial procedures and outcome measurement. Any clinical outcomes are therefore presented as exploratory and descriptive findings intended to inform a future definitive trial. |
| **Reviewer #2: The paper is original and the aim is clear. Here are some recommendations that may be taken into consideration:** |  |
| 1. In "Data Availability" section in submission file, the sentence is copied but lacks the corresponding URL (then another section mentions that it will be present upon acceptance). Please do not forget to modify sentences | We thank the reviewer for these comments and have amended the sentence to indicated ‘data availability’ will be made upon request. |
| 2. There are some typos; and some sentence structure can be enhanced to maintain the reading flow. | Thank you for this comment. We have carefully proofread the manuscript and corrected a number of typographical, grammatical and formatting errors. We have also revised several sentences throughout the manuscript to improve clarity, readability and overall flow, while ensuring that the meaning and interpretation of the findings remain unchanged. |
| 3. This study has a previous published protocol. Please state that explicitly. | This has now been stated in the paper. |
| 4. Also, please "unblind" reference 31 | Reference added. |
| 5. Based on that, the introduction and method section should be shorter, more direct and concise; thus, reducing redundancy (including the figure) compared to the published protocol. In addition, the introduction can be restructured since now it reads as listing (somehow) with so many subsections. | We thank the reviewer for this suggestion. We carefully considered shortening and restructuring the Introduction and Methods sections. However, we elected to retain the current structure because the MATILDA intervention is a complex intervention and the feasibility trial incorporated several components, including feasibility outcomes, process evaluation, economic evaluation, and RE-AIM implementation outcomes. We believe the current level of detail is necessary to provide readers with sufficient context to understand the intervention, trial procedures, and interpretation of findings. Although a study protocol has been published, the results paper is intended to be a standalone manuscript and therefore requires key methodological and contextual information to be presented within the paper itself. We have reviewed these sections for clarity but have not made substantial structural changes. |
| 6. Also, the paper can benefit in these sections to state what/how differences from the protocol are present/managed in this paper. | We thank the reviewer for this helpful suggestion. The trial was conducted largely as described in the published protocol. We have reviewed the manuscript and now explicitly state that the study was undertaken in accordance with the published protocol. In addition, we have identified and described the small number of operational changes that occurred during trial delivery (e.g., modifications to outcome measure administration and reporting of results). None of these changes altered the feasibility objectives, intervention content, randomisation procedures, or primary study outcomes. |
| 7. in the figure, it mentions 100 participants initially but in text it mentions 95. Also, many numbers throughout the manuscript need organizing, rechecking or clarifications. Also, better to state % referring to the baseline total (as an example but not limited to this only: it is more inferential to see % of participants that are still in the study from t=0 reaching 6 months and 12 months and not to calculate % of participants at 12 months referring to 6 months number or at least mention both or clarify). Also, in abstract there is 89% while in results it is 60% (calculated % from different target/total), so at least this also may need reframing or modification. Then, in recruitment it is stated 95% (of target 64 while it is 89% as in abstract). | Thank you for highlighting these inconsistencies. We carefully reviewed all participant numbers and percentages reported throughout the manuscript. The discrepancy between the participant flow diagram and text has been corrected, and all recruitment and retention figures now align. We have standardised the reporting of percentages and clearly identified denominators throughout the manuscript. Recruitment is now consistently reported as 57 of the target 64 participants (89%), while retention percentages are calculated from the baseline recruited sample unless otherwise specified. The Abstract, Results section, tables and figure have all been checked and revised to ensure consistency and clarity. |
| 8. Even if no sample size calculations, choice of 64 can be more justified. | Thank you for this comment. As this was a feasibility trial, a formal sample size calculation was not undertaken. The target sample size of 64 participants was selected for pragmatic reasons based on the capacity of the Volunteer Co-ordinator to recruit, manage and support older adults with intellectual disability participating in the community groups across the two study sites. The sample size was considered sufficient to assess key feasibility outcomes, including recruitment, retention, intervention adherence and data collection procedures, and to inform the design of a future definitive trial. |
| 9. For table 1, please check numbers and %s. In control group/gender, the total % is >100. The percentage values have been removed. Also, for Age, I believe, since it is SD, it should be (7.1 not 7.1%) The % value has been removed. | Thank you for identifying these errors. We reviewed Table 1 and found that the gender percentages in the control group were incorrectly reported, resulting in a total exceeding 100%. The percentage values have now been corrected/removed as appropriate. In addition, the age variable was reported as a mean (SD), and the erroneous percentage symbol attached to the standard deviation value has been removed. Table 1 has been checked carefully to ensure all figures are accurate and internally consistent. |
| 10. For collected data on health, are all the listed diseases self-reported? please add clarification or the sentence may need modification. | Yes, health data is all self-reported this has been clarified in the methods section. |
| 11. for Table 2, better not to mention the two numbers 28 and 25, i think it is enough to state 25 while already explaining it in text (and abstract). In the abstract the figure is 28! | We thank the reviewer for these comments, and these amendments have been made in Table 2 and abstract. |
| 12. For table 3, the column (other) better to be named (excluded) and to enhance readability, it is better to be placed after (total) column. | We thank the reviewer for these comments, these amendments have been made in Table 3. |
| 13. For some clinical outcomes, it is more inferential to compare the difference between 6 and 12 months with respect to control group. Please clarify or emphasize. | This comparison was made in order to have individuals act as their own controls. In addition, an inter-group comparison has also been made to establish a theoretical statistic power working on the values shown in Table 4 for the measure of anxiety. The mean difference, at the third point in time has approximately a 2-point difference for the measure of anxiety. The data in Table 4 also shows a smaller standard deviation for the Matilda group on the third occasion; hence a greater homogeneity in the scores, which might be expected where a group had a better performance (11.2 SD vs 9.1 SD.) over time. For data with these expected values a sample of just over 300 participants would be required to reliability detected such a difference with a power of 0.8.  *“Table 4 shows that the Glasgow Anxiety Scale scores for the participants in the MATILDA arm decreased from a mean of 20.7 at baseline to a mean of 18.1 at 6-months and a mean of 16 at 12-months. This is compared to the participants in the control arm who displayed a slight decrease across the three time-points (baseline mean (19.6), 6-month mean (17.1) and 12-month mean (18.2)).”* |
| 15. Even if exploratory, comparison measures between groups may be considered (effect sizes, difference, confidence intervals). Also, some clinical outcomes can be discussed more (suggest solutions, mention reasons, explain results and.....) | The major effect is in relation to the Glasgow Anxiety Inventory: see above comments. |
| 16. Discussion can benefit more from clearer limitations (as generalizability in different populations, duration of control group which may not be comparable, mentors did not complete the scoring....) and future research | Thank you for this helpful suggestion. We have expanded the Discussion section to provide a more comprehensive consideration of the study limitations and implications for future research. Specifically, we have acknowledged the limited generalisability of findings beyond the two UK study sites and the relatively small feasibility sample. We have also discussed the potential impact of the different duration and nature of the control condition, as well as limitations associated with incomplete mentor-reported outcome data. Finally, we have expanded the section on future research to outline the requirements for a definitive trial, including a larger and more diverse sample, longer-term follow-up, and further evaluation of clinical, implementation and economic outcomes. |
| **Reviewer #3: Thank you for the opportunity to review this valuable article. The article is a good one, and the study addresses an important gap in the literature, as older adults with intellectual disabilities remain an underserved population with limited opportunities for community engagement and social participation. My suggestions are as follows:** |  |
| 1- The introduction should more thoroughly address the challenges and issues in this field, adopt a more international approach, and discuss possible strategies to address them. | Thank you for this suggestion. We agree that the challenges facing older adults with intellectual disability are important and were considered in developing the study rationale. However, given the word limit constraints and the existence of a previously published study protocol that provides more detailed background information, we have elected to retain the current introduction. We believe the introduction provides sufficient context for the aims of the present paper, which focuses on reporting the findings of a feasibility randomised controlled trial and process evaluation rather than providing a comprehensive review of the international literature. |
| 2- The authors should clarify the Progression Criteria specifically, whether recruitment, retention, adherence, and acceptability thresholds were established a priori. And how were decisions regarding progression to a definitive trial determined? Were all feasibility objectives achieved according to predefined benchmarks? | Thank you for this comment. Progression criteria relating to recruitment, retention, intervention adherence and acceptability were pre-specified in the study protocol. We have clarified these criteria in the Methods section and have reported the extent to which each criterion was achieved in the Results section. Overall, the findings indicated that the predefined feasibility objectives were achieved and supported progression to a future definitive trial, subject to refinements identified through the process evaluation. |
| 3- In "the methodology, " the lack of transparency in some areas makes it difficult to evaluate internal validity. The authors should report the following details explicitly: a) Method used for sequence generation. b) Allocation concealment procedures. c) Individual versus cluster randomization considerations. d) Any stratification or blocking procedures employed. | We thank the reviewer for these comments, reviewer 1 as raised similar comments above, these amendments have been addressed in the amended paper. |
| 4-Sample Size should be justified by the authors, and the rationale for selecting the target sample size should be described more thoroughly. | We thank the reviewer for these comments, reviewer 2 as raised similar comments, these amendments have been addressed in the amended paper. |
| 5-The authors have to report descriptively, including Participant demographics, disability characteristics, baseline outcome measures, and any notable imbalances should be acknowledged and discussed. | Thank you for this comment. Participant demographics, disability characteristics and baseline outcome measures are presented in Table 1. We have revised the Results section to more clearly describe the baseline characteristics of the two groups and to acknowledge any notable imbalances observed at baseline. As expected in a feasibility trial with a relatively small sample, some differences were evident between groups; however, these were not considered sufficiently large to affect the assessment of feasibility outcomes. |
| 6- The authors should include a summary table comparing targets versus achieved outcomes, which would substantially strengthen the feasibility conclusions. | Thank you for this suggestion. We considered adding a summary table comparing targets and achieved outcomes. However, as the feasibility outcomes are already reported in detail within the Results section and associated tables, we felt that an additional summary table would largely duplicate existing information. We have therefore retained the current presentation, which we believe provides a clear account of the study's feasibility findings. |
| 7- In the "discussion," the following should be revised to ensure that: Statistical findings are interpreted cautiously. Any observed changes are clearly described as exploratory, and readers are not led to infer intervention efficacy beyond the scope of a feasibility study. Furthermore, the distinction between feasibility outcomes and effectiveness outcomes should be more consistently emphasized throughout the manuscript. | Thank you for this comment. We agree that findings relating to clinical outcomes should be interpreted cautiously. We have revised the Discussion to emphasise that the primary purpose of this study was to assess feasibility and acceptability rather than intervention effectiveness. We have clarified that any observed changes in clinical outcomes are exploratory in nature and that the study was not powered to detect statistically significant differences between groups. Throughout the manuscript, we have strengthened the distinction between feasibility outcomes (e.g., recruitment, retention, adherence and acceptability) and effectiveness outcomes. We have also revised the language used when discussing clinical findings to avoid implying efficacy and to acknowledge that a fully powered trial is required to determine intervention effectiveness. |
| 8- For better "Literature Contextualization," the authors should bring more from literature to have a more detailed comparison with previous interventions designed to reduce loneliness, increase community participation, or promote social inclusion among adults with intellectual disabilities. I think this would help readers better understand the contribution of MATILDA relative to existing evidence. | We thank the reviewer for this helpful suggestion. We acknowledge that there is a substantial international literature examining interventions designed to reduce loneliness, enhance social inclusion, and increase community participation both within the general population and among people with intellectual disabilities. We carefully considered expanding the literature contextualisation within the Discussion. However, given the journal's word limit and the primary aim of this paper to report the feasibility, acceptability, and implementation findings of the MATILDA intervention, we sought to maintain a balance between contextualisation and reporting of the study findings. We believe that the current discussion provides sufficient reference to the most relevant literature to situate MATILDA within the existing evidence base while allowing adequate space to present the feasibility outcomes of this complex intervention. Nevertheless, we have reviewed and strengthened the Discussion where possible to further highlight how MATILDA contributes to the existing literature on loneliness, social inclusion, and community participation among adults with intellectual disabilities. |
| 9- The economic evaluation could be developed further in the discussion, and the authors may wish to elaborate on the Feasibility of collecting resource-use data, Potential cost drivers, and implications for a future cost-effectiveness analysis. | We thank the reviewer for these comments, these amendments have been made in the Results seetion. |
