# Supplementary material for: Implementing the MATILDA intervention to enhance community participation for older adults with intellectual disability: A feasibility randomised controlled trial and RE-AIM guided process evaluation
Source: PLoS One. 2026 Sep 22;21(9):e0341940. doi: 10.1371/journal.pone.0341940 (PMC13596784; doi:10.1371/journal.pone.0341940)
Supplement: Peer review history

# Peer review: Decision Letter 0

**DOI:** 10.1371/journal.pone.0341940.r001

**Authors:** Mukhtiar Baig

16 Jun 2026

Dear Dr. Taggart,

Thank you for submitting your manuscript to PLOS ONE. After careful consideration, we feel that it has merit but does not fully meet PLOS ONE’s publication criteria as it currently stands. Therefore, we invite you to submit a revised version of the manuscript that addresses the points raised during the review process.

plosone@plos.org. When you're ready to submit your revision, log on to https://www.editorialmanager.com/pone/ and select the 'Submissions Needing Revision' folder to locate your manuscript file.

- A letter that responds to each point raised by the academic editor and reviewer(s). You should upload this letter as a separate file labeled 'Response to Reviewers'.
- A marked-up copy of your manuscript that highlights changes made to the original version. You should upload this as a separate file labeled 'Revised Manuscript with Track Changes'.
- An unmarked version of your revised paper without tracked changes. You should upload this as a separate file labeled 'Manuscript'.

If you would like to make changes to your financial disclosure, please include your updated statement in your cover letter. Guidelines for resubmitting your figure files are available below the reviewer comments at the end of this letter.

If applicable, we recommend that you deposit your laboratory protocols in protocols.io to enhance the reproducibility of your results. Protocols.io assigns your protocol its own identifier (DOI) so that it can be cited independently in the future. For instructions see: https://journals.plos.org/plosone/s/submission-guidelines#loc-laboratory-protocols. Additionally, PLOS ONE offers an option for publishing peer-reviewed Lab Protocol articles, which describe protocols hosted on protocols.io. Read more information on sharing protocols at https://plos.org/protocols?utm_medium=editorial-email&utm_source=authorletters&utm_campaign=protocols.

As the corresponding author, your ORCID iD is verified in the submission system and will appear in the published article. PLOS supports the use of ORCID, and we encourage all coauthors to register for an ORCID iD and use it as well. Please encourage your coauthors to verify their ORCID iD within the submission system before final acceptance, as unverified ORCID iDs will not appear in the published article. *Only*  the individual author can complete the verification step; PLOS staff *cannot*  verify ORCID iDs on behalf of authors.

We look forward to receiving your revised manuscript.

Kind regards,

Mukhtiar Baig, Ph.D.

Academic Editor

PLOS One

Journal requirements:

When submitting your revision, we need you to address these additional requirements.

1. Please ensure that your manuscript meets PLOS ONE's style requirements, including those for file naming. The PLOS ONE style templates can be found at

https://journals.plos.org/plosone/s/file?id=wjVg/PLOSOne_formatting_sample_main_body.pdf and

https://journals.plos.org/plosone/s/file?id=ba62/PLOSOne_formatting_sample_title_authors_affiliations.pdf

2. Thank you for stating the following financial disclosure:

“This study was funded by the National Institute for Health and Care Research (NIHR) Public Health Research (PHR) Programme (NIHR129125). The views expressed are those of the authors and not necessarily those of the NIHR or the Department of Health and Social Care.”

Please state what role the funders took in the study.  If the funders had no role, please state: "The funders had no role in study design, data collection and analysis, decision to publish, or preparation of the manuscript."

If this statement is not correct you must amend it as needed.

Please include this amended Role of Funder statement in your cover letter; we will change the online submission form on your behalf.

3. Thank you for stating the following in the Acknowledgments Section of your manuscript:

“This study was funded by the National Institute for Health and Care Research (NIHR) Public Health Research (PHR) Programme (NIHR129125). The views expressed are those of the authors and not necessarily those of the NIHR or the Department of Health and Social Care.”

We note that you have provided funding information that is currently declared in your Funding Statement. However, funding information should not appear in the Acknowledgments section or other areas of your manuscript. We will only publish funding information present in the Funding Statement section of the online submission form.

Please remove any funding-related text from the manuscript and let us know how you would like to update your Funding Statement. Currently, your Funding Statement reads as follows:

“This study was funded by the National Institute for Health and Care Research (NIHR) Public Health Research (PHR) Programme (NIHR129125). The views expressed are those of the authors and not necessarily those of the NIHR or the Department of Health and Social Care.”

Please include your amended statements within your cover letter; we will change the online submission form on your behalf.

4. When completing the data availability statement of the submission form, you indicated that you will make your data available on acceptance. We strongly recommend all authors decide on a data sharing plan before acceptance, as the process can be lengthy and hold up publication timelines. Please note that, though access restrictions are acceptable now, your entire data will need to be made freely accessible if your manuscript is accepted for publication. This policy applies to all data except where public deposition would breach compliance with the protocol approved by your research ethics board. If you are unable to adhere to our open data policy, please kindly revise your statement to explain your reasoning and we will seek the editor's input on an exemption. Please be assured that, once you have provided your new statement, the assessment of your exemption will not hold up the peer review process.

5. Please include a separate caption for each figure in your manuscript.

If the reviewer comments include a recommendation to cite specific previously published works, please review and evaluate these publications to determine whether they are relevant and should be cited. There is no requirement to cite these works unless the editor has indicated otherwise.

[Note: HTML markup is below. Please do not edit.]

Reviewers' comments:

Reviewer's Responses to Questions

**Comments to the Author**

1. Is the manuscript technically sound, and do the data support the conclusions?

Reviewer #1: Partly

Reviewer #2: Partly

Reviewer #3: Yes

**********

2. Has the statistical analysis been performed appropriately and rigorously?

Reviewer #1: No

Reviewer #2: N/A

Reviewer #3: Yes

**********

3. Have the authors made all data underlying the findings in their manuscript fully available?

The PLOS Data policy

Reviewer #1: Yes

Reviewer #2: No

Reviewer #3: Yes

**********

4. Is the manuscript presented in an intelligible fashion and written in standard English?

Reviewer #1: No

Reviewer #2: Yes

Reviewer #3: Yes

**********

Reviewer #1: The primary objective of this randomized two arm feasibility study was to assess the practicality of conducting the trial and to undertake a RE AIM guided process evaluation of the MATILDA intervention, delivered across two UK sites with 1:1 allocation to MATILDA plus usual care versus an active control plus usual care. The topic is important, the investigators completed a multisite feasibility randomized study in an underserved population, and the protocol includes many of the operational elements expected for this design. However, there are several major trial design and statistical concerns.

Statistical critiques:

1. The manuscript does not present a clear hierarchy that keeps the primary feasibility aim front and center, because feasibility outcomes, clinical outcomes, process evaluation, and economic elements are all interwoven without a crisp primary feasibility analysis narrative; the authors should tighten the paper around prespecified feasibility endpoints and progression criteria, and keep secondary and exploratory outcomes clearly labeled and clearly subordinated to the feasibility aim.

2. The blinding description is internally inconsistent, because the trial is described as single blind while the research staff conducting baseline and follow up assessments were not blinded and participant and mentor blinding was not possible; the authors should describe the study as it was conducted, clarify what protections were used to reduce assessment bias, and ensure Methods and Limitations use consistent language.

3. The randomization reporting is incomplete for reproducibility even though stratification by the two sites is stated, because key details such as the allocation concealment mechanism and the permuted block approach with mixed block sizes described in the protocol are not fully carried into the manuscript; the authors should report sequence generation and concealment clearly, including who generated the sequence, how centralized allocation was implemented, and the block structure description at a level that preserves concealment.

4. The two arm design is not used analytically in a way that justifies its inclusion, because the results are primarily descriptive summaries within arms and across time points with limited direct estimation of between arm contrasts; the authors should explain why an active control arm was required for this feasibility question and then report between arm differences for key outcomes at 6 and 12 months, at minimum as estimated effects with 95% confidence intervals.

5. Table 4 and other longitudinal outcome summaries do not provide statistical inference, which makes it difficult for readers to interpret whether observed changes or apparent differences are potentially meaningful versus expected variability in a feasibility sample; the authors should add 95% confidence intervals for within arm change from baseline and for between arm differences at each follow up time point, and they may use a simple baseline adjusted ANCOVA or a repeated measures mixed effect model without overemphasizing p-values.

6. The analysis plan is not presented in a prespecified, verifiable way in the manuscript, because the paper states that no formal statistical tests were performed and does not clearly specify how site stratification is handled in analyses, how missing data are addressed, or how multiple secondary and exploratory outcomes are managed; the authors should either provide a standalone SAP as a supplement or map the protocol statistical methods into the manuscript with enough specificity that the reader can see what was planned versus exploratory.

7. The manuscript describes modifications to at least one instrument during follow-up, which can compromise comparability across time; the authors should specify when changes occurred, how scoring and interpretation remain comparable to baseline, and what sensitivity analyses support longitudinal conclusions under the modified measurement approach.

Reviewer #2: The paper is original and the aim is clear. Here are some recommendations that may be taken into consideration:

1. In "Data Availability" section in submission file, the sentence is copied but lacks the corresponding URL (then another section mentions that it will be present upon acceptance). Please do not forget to modify sentences

2. There are some typos; and some sentence structure can be enhanced to maintain the reading flow.

3. This study has a previous published protocol. Please state that explicitly.

4. Also, please "unblind" reference 31

5. Based on that, the introduction and method section should be shorter, more direct and concise; thus, reducing redundancy (including the figure) compared to the published protocol. In addition, the introduction can be restructured since now it reads as listing (somehow) with so many subsections.

6. Also, the paper can benefit in these sections to state what/how differences from the protocol are present/managed in this paper.

7. in the figure, it mentions 100 participants initially but in text it mentions 95. Also, many numbers throughout the manuscript need organizing, rechecking or clarifications. Also, better to state % referring to the baseline total (as an example but not limited to this only: it is more inferential to see % of participants that are still in the study from t=0 reaching 6 months and 12 months and not to calculate % of participants at 12 months referring to 6 months number or at least mention both or clarify). Also, in abstract there is 89% while in results it is 60% (calculated % from different target/total), so at least this also may need reframing or modification. Then, in recruitment it is stated 95% (of target 64 while it is 89% as in abstract).

8. Even if no sample size calculations, choice of 64 can be more justified.

9. For table 1, please check numbers and %s. In control group/gender, the total % is >100. Also, for Age, I believe, since it is SD, it should be (7.1 not 7.1%)

10. For collected data on health, are all the listed diseases self-reported? please add clarification or the sentence may need modification

11. for Table 2, better not to mention the two numbers 28 and 25, i think it is enough to state 25 while already explaining it in text (and abstract)

12. For table 3, the column (other) better to be named (excluded) and to enhance readability, it is better to be placed after (total) column.

13. For some clinical outcomes, it is more inferential to compare the difference between 6 and 12 months with respect to control group. Please clarify or emphasize.

14. for table 4, in the first column, the total score may be added (to facilitate reading of score and to know out of how much, especially there was modifications adopted)

15. even if exploratory, comparison measures between groups may be considered (effect sizes, difference, confidence intervals). Also, some clinical outcomes can be discussed more (suggest solutions, mention reasons, explain results and.....)

16. discussion can benefit more from clearer limitations (as generalizability in different populations, duration of control group which may not be comparable, mentors did not complete the scoring....) and future research

Reviewer #3: Thank you for the opportunity to review this valuable article.

This article reports a feasibility randomized controlled trial evaluating the implementation and acceptability of the MATILDA intervention, a community participation program for older adults with intellectual disabilities. The article is a good one, and the study addresses an important gap in the literature, as older adults with intellectual disabilities remain an underserved population with limited opportunities for community engagement and social participation. My suggestions are as follows:

1- The introduction should more thoroughly address the challenges and issues in this field, adopt a more international approach, and discuss possible strategies to address them.

2- The authors should clarify the Progression Criteria specifically, whether recruitment, retention, adherence, and acceptability thresholds were established a priori. And how were decisions regarding progression to a definitive trial determined? Were all feasibility objectives achieved according to predefined benchmarks?

3- In "the methodology, " the lack of transparency in some areas makes it difficult to evaluate internal validity. The authors should report the following details explicitly:

a) Method used for sequence generation. b) Allocation concealment procedures. c) Individual versus cluster randomization considerations. d) Any stratification or blocking procedures employed.

4-Sample Size should be justified by the authors, and the rationale for selecting the target sample size should be described more thoroughly.

5-The authors have to report descriptively, including Participant demographics, disability characteristics, baseline outcome measures, and any notable imbalances should be acknowledged and discussed.

6- The authors should include a summary table comparing targets versus achieved outcomes, which would substantially strengthen the feasibility conclusions.

7- In the "discussion," the following should be revised to ensure that: Statistical findings are interpreted cautiously.

Any observed changes are clearly described as exploratory, and readers are not led to infer intervention efficacy beyond the scope of a feasibility study. Furthermore, the distinction between feasibility outcomes and effectiveness outcomes should be more consistently emphasized throughout the manuscript.

8- For better "Literature Contextualization," the authors should bring more from literature to have a more detailed comparison with previous interventions designed to reduce loneliness, increase community participation, or promote social inclusion among adults with intellectual disabilities. I think this would help readers better understand the contribution of MATILDA relative to existing evidence.

9- The economic evaluation could be developed further in the discussion, and the authors may wish to elaborate on the Feasibility of collecting resource-use data, Potential cost drivers, and implications for a future cost-effectiveness analysis.

**********

what does this mean?). If published, this will include your full peer review and any attached files.

If you choose “no”, your identity will remain anonymous but your review may still be made public.

**Do you want your identity to be public for this peer review?**  For information about this choice, including consent withdrawal, please see our Privacy Policy

Reviewer #1: No

Reviewer #2: No

Reviewer #3: No

**********

[NOTE: If reviewer comments were submitted as an attachment file, they will be attached to this email and accessible via the submission site. Please log into your account, locate the manuscript record, and check for the action link "View Attachments". If this link does not appear, there are no attachment files.]

To ensure your figures meet our technical requirements, please review our figure guidelines: https://journals.plos.org/plosone/s/figures

You may also use PLOS’s free figure tool, NAAS, to help you prepare publication quality figures: https://journals.plos.org/plosone/s/figures#loc-tools-for-figure-preparation.

NAAS will assess whether your figures meet our technical requirements by comparing each figure against our figure specifications.

# Author comment: Author response to Decision Letter 1

**DOI:** 10.1371/journal.pone.0341940.r002

27 Jul 2026

Attached a response to the reviewers comments

Attachment Submitted filename: MATILDA RESPONSE 3rd July 2026.docx

# Peer review: Decision Letter 1

**DOI:** 10.1371/journal.pone.0341940.r003

**Authors:** Mukhtiar Baig

11 Aug 2026

Implementing the MATILDA intervention to enhance community participation for older adults with intellectual disability: a feasibility randomised controlled trial and RE-AIM guided process evaluation

PONE-D-26-01629R1

Dear Dr. Taggart,

We’re pleased to inform you that your manuscript has been judged scientifically suitable for publication and will be formally accepted for publication once it meets all outstanding technical requirements.

Within one week, you’ll receive an e-mail detailing the required amendments. When these have been addressed, you’ll receive a formal acceptance letter and your manuscript will be scheduled for publication.

An invoice will be generated when your article is formally accepted. Please note, if your institution has a publishing partnership with PLOS and your article meets the relevant criteria, all or part of your publication costs will be covered. Please make sure your user information is up-to-date by logging into Editorial Manager at Editorial Manager® and clicking the ‘Update My Information' link at the top of the page. For questions related to billing, please contact billing support.

If your institution or institutions have a press office, please notify them about your upcoming paper to help maximize its impact. If they’ll be preparing press materials, please inform our press team as soon as possible -- no later than 48 hours after receiving the formal acceptance. Your manuscript will remain under strict press embargo until 2 pm Eastern Time on the date of publication. For more information, please contact onepress@plos.org.

Kind regards,

Mukhtiar Baig, Ph.D.

Academic Editor

PLOS One

Reviewers' comments:

Reviewer's Responses to Questions

**Comments to the Author**

Reviewer #1: All comments have been addressed

**********

2. Is the manuscript technically sound, and do the data support the conclusions?

Reviewer #1: Yes

**********

3. Has the statistical analysis been performed appropriately and rigorously?

Reviewer #1: Yes

**********

4. Have the authors made all data underlying the findings in their manuscript fully available?

The PLOS Data policy

Reviewer #1: Yes

**********

5. Is the manuscript presented in an intelligible fashion and written in standard English?

Reviewer #1: Yes

**********

Reviewer #1: The authors have responded adequately to my statistical comments, and the manuscript has significantly improved.

**********

what does this mean?). If published, this will include your full peer review and any attached files.

If you choose “no”, your identity will remain anonymous but your review may still be made public.

**Do you want your identity to be public for this peer review?**  For information about this choice, including consent withdrawal, please see our Privacy Policy

Reviewer #1: No

**********

# Editor report: Acceptance letter

**DOI:** 10.1371/journal.pone.0341940.r004

**Authors:** Mukhtiar Baig

PONE-D-26-01629R1

PLOS One

Dear Dr. Taggart,

I'm pleased to inform you that your manuscript has been deemed suitable for publication in PLOS One. Congratulations! Your manuscript is now being handed over to our production team.

At this stage, our production department will prepare your paper for publication. This includes ensuring the following:

* All references, tables, and figures are properly cited

* All relevant supporting information is included in the manuscript submission,

* There are no issues that prevent the paper from being properly typeset

You will receive further instructions from the production team, including instructions on how to review your proof when it is ready. Please keep in mind that we are working through a large volume of accepted articles, so please give us a few days to review your paper and let you know the next and final steps.

Lastly, if your institution or institutions have a press office, please let them know about your upcoming paper now to help maximize its impact. If they'll be preparing press materials, please inform our press team within the next 48 hours. Your manuscript will remain under strict press embargo until 2 pm Eastern Time on the date of publication. For more information, please contact onepress@plos.org.

You will receive an invoice from PLOS for your publication fee after your manuscript has reached the completed accept phase. If you receive an email requesting payment before acceptance or for any other service, this may be a phishing scheme. Learn how to identify phishing emails and protect your accounts at https://explore.plos.org/phishing.

If we can help with anything else, please email us at customercare@plos.org.

Thank you for submitting your work to PLOS One and supporting open access.

Kind regards,

PLOS One Editorial Office Staff

on behalf of

Professor Mukhtiar Baig

Academic Editor

PLOS One
